# Transcriptomic Analysis of Seed Germination Under Salt Stress in Two Desert Sister Species (*Populus euphratica* and *P. pruinosa*)

**DOI:** 10.3389/fgene.2019.00231

**Published:** 2019-03-25

**Authors:** Caihua Zhang, Wenchun Luo, Yanda Li, Xu Zhang, Xiaotao Bai, Zhimin Niu, Xiao Zhang, Zhijun Li, Dongshi Wan

**Affiliations:** ^1^State Key Laboratory of Grassland Agro-Ecosystem, School of Life Sciences, Lanzhou University, Lanzhou, China; ^2^Computer Science and Engineering Department, University of California, San Diego, La Jolla, CA, United States; ^3^Xinjiang Production & Construction Corps, Key Laboratory of Protection and Utilization of Biological Resources in Tarim Basin, College of Life Sciences, Tarim University, Xinjiang, China

**Keywords:** transcriptome, salt stress, seed germination, differentially expressed gene, desert poplar species

## Abstract

As a major abiotic stress, soil salinity limits seed germination and plant growth, development and production. Seed germination is highly related not only to the seedlings survival rate but also subsequent vegetative growth. *Populus euphratica* and *P. pruinosa* are closely related species that show a distinguished adaptability to salinity stress. In this study, we performed an integrative transcriptome analyses of three seed germination phases from *P. euphratica* and *P. pruinosa* under salt stress. A two-dimensional data set of this study provides a comprehensive view of the dynamic biochemical processes that underpin seed germination and salt tolerance. Our analysis identified 12831 differentially expressed genes (DEGs) for seed germination processes and 8071 DEGs for salt tolerance in the two species. Furthermore, we identified the expression profiles and main pathways in each growth phase. For seed germination, a large number of DEGs, including those involved in energy production and hormonal regulation pathways, were transiently and specifically induced in the late phase. In the comparison of salt tolerance between the two species, the flavonoid and brassinosteroid pathways were significantly enriched. More specifically, in the flavonoid pathway, *FLS* and *F3*′*5*′*H* exhibited significant differential expression. In the brassinosteroid pathway, the expression levels of *DWF4*, *BR6OX2* and *ROT3* were notably higher in *P. pruinosa* than in *P. euphratica*. Our results describe transcript dynamics and highlight secondary metabolite pathways involved in the response to salt stress during the seed germination of two desert poplars.

## Introduction

Soil salinization is caused by many factors and conditions, such as unsuitable irrigation practices, irrigation with salinized water and seasonal effects (Ottow et al., 2005; Annunziata et al., 2017). As one of the most prominent abiotic stresses, salinity stress is considered the greatest threat to crop production and environmental conservation (Ottow et al., 2005; Arbona et al., 2013). Salinity stress leads to osmotic and ionic stress, which reduces cell and tissue expansion, and to ion excesses that changes the osmotic potential of plant cells and induce nutritional imbalances (Munns, 2002), sequentially affecting plant growth, development and survival (Carillo et al., 2019). To solve the serious problem of soil salinization, various efforts have been made; these efforts mainly concentrate on enhancing the salt resistance of economically important salt-sensitive plants through traditional breeding and biotechnological approaches or the use of plants that naturally display high salt tolerance (Flowers, 2004).

*Populus euphratica* and its sister species *P. pruinosa* are naturally distributed in China’s western desert region; due to their extraordinary adaptability to desert environments (Chen et al., 2002; Hukin et al., 2005), both species are also called desert poplars. The distinguished adaptability of these species provides beneficial ecological effects in northwest China. Currently, both poplars are considered important genetic resources in tree breeding and in research elucidating physiological and molecular mechanisms involving stress tolerance in trees (Tuskan et al., 2006; Wullschleger et al., 2013). As the genome data of *P. euphratica* becomes available (Ma et al., 2013), the resistance mechanism of both poplars have been revealed at multiple levels, e.g., a phylogenetic analysis shows that the two species diverged approximately 1–2 million years ago (Wang J. et al., 2011), and ancient polymorphisms contributed to their genomic divergence (Ma et al., 2018). In addition to leaf morphology and leaf trichome differences (Ma et al., 2016), both poplars occupy different ecological habitats. *P. pruinosa* prefers desert areas with high ground water levels, while *P. euphratica* can grow in desert areas where the groundwater levels are low (Ottow et al., 2005). These differences between the sister poplars result from differences in genetic mechanisms, such as the adaptive evolution of genes (Ma et al., 2013; Zhang et al., 2014) and gene expression divergences among orthologs (Qiu et al., 2011; Zhang et al., 2013).

Seed germination is constrained significantly by soil salinity (Kaya et al., 2003). Soil salinity creates an osmotic potential around the outside of seeds, resulting in decreased water uptake during germination and an increase in the excessive uptake of ions, which causes the toxic effects of Na^+^ and Cl^-^ ions to seeds (Murillo-Amador et al., 2002; Khajeh-Hosseini et al., 2003). Therefore, salt stress can inhibit or delay seed germination (Almansouri et al., 2001). However, studies focusing on the genetic mechanism of seed germination under salt stress are limited.

Seed germination begins with imbibition and ends with the embryonic axis breaking through the seed coat (Bewley et al., 2012). Seed germination includes three phases. In phase I, the seed begins to expand, with a rapidly increasing water content. Then, the seed enters a plateau phase (phase II), in which the water uptake remains at a stable level. In phase III, the water uptake increases rapidly. Phase III ceases as the embryonic axis breaks through the seed coat, upon which seed germination is complete (Bewley, 1997). Energy production and respiration play important roles in the seed germination process. In the early stage, anaerobic respiration provides the main energy source, and then respiratory activity increases with oxygen uptake. Subsequently, plant hormones, such as gibberellins (GA), abscisic acid (ABA), brassinosteroids (BRs), ethylene, auxins, and cytokinins, are widely involved in determining the physiological state of a seed and regulating the germination process (Kucera et al., 2005; Holdsworth et al., 2008; Müller et al., 2009; North et al., 2010). Furthermore, numerous complex networks, including those related to gene expression and regulation commanded by various transcription factors (Chen et al., 2002), ion transporting processes, such as NHX (Na^+^/H^+^ antiporter), SOS (salt overly sensitive) (Zhu, 2001), and HKT (high-affinity K^+^ transporter) (Ren et al., 2005) processes, and secondary metabolism are all involved in the response to salt stress (Bewley, 1982, 1997; Bewley and Black, 1984; Biligetu et al., 2011). Recently, transcriptomic analyses of several poplar species under various stresses have been extensively conducted (Chen et al., 2002, 2012; Brinker et al., 2010; Janz et al., 2010; Qiu et al., 2011; Wang J. et al., 2011; Ma et al., 2013; Ziemann et al., 2013). These data provide us with a basic understanding of seed germination. However, detailed transcriptomic dynamics and physiological mechanisms under salt stress during seed germination have not yet been revealed. Such an exploration might be useful to identify the genes that improve poplar salt tolerance by biotechnological manipulation. Moreover, most genes associated with seed germination are poorly understood due to the complexity of the germination process.

Here, we present a comprehensive transcriptome study encompassing the whole process of seed germination for two species under salt stress, which provides a valuable gene resource for genetic manipulation in poplar breeding.

## Materials and Methods

### Plant Materials and Growth Conditions

We collected three replicate samples of the seeds of the two studied species from a total of 18 trees in the Tarim Basin (Xinjiang, China) and stored the seeds at 4°C. For germination, vigorous seeds were imbibed in distilled water (control), 0.2%, 0.4%, 0.6%, 0.8%, 1.0%, 1.2%, 1.4%, and 1.6% NaCl, and then germinated on wet filter paper in 9 cm diameter Petri dishes in a plant growth incubator (21°C 200 μmol m^-2^s^-1^, 16 h: 8 h light/dark photoperiod). The germination rate was measured using the Chinese national standard test (GB2772-1999). Each sample contained 50 seeds and had three replicates (Wang et al., 2013). The germinating seeds were scanned and photographed using a stereo microscope (Nikon SM Z1500, Japan) to record their morphology. The moisture content of the seed samples was measured in seeds oven dried at 75°C to a constant weight. The moisture content [g (g FW)^-1^] was calculated as [(FW-DW)/FW].

The percentage of seeds with two cotyledons turning green or with emerging radicles (>1 mm) was considered the germination rate (Wang Y. et al., 2011). For the germination percentage, counts were made until no additional germination was observed for 72 h (Bradford, 1990). To elucidate the threshold salinity for the two species under the salt treatments, we measured relative indexes, including GR, RGP, GT, GI, K, and RSH (Imit et al., 2015). For RNA isolation, the seeds were imbibed in 1.0% NaCl (to expose them to salt stress) and then removed after 4, 12, 24, 48, and 72 h for RNA preparation. The control samples were collected from dry seeds (0 h). We rapidly transferred all the samples to storage at -80°C before RNA extraction.

### Reactive Oxygen Species (ROS) Level and Enzyme Activity Determination

For germination, seeds were imbibed in 0%, 0.4%, 0.8% and 1.0% NaCl as described above for 24 h. The levels of ROS, superoxide dismutase (SOD) and catalase (CAT) were measured using the standard protocol for the toolkit from Suzhou Comin Biotechnology.

### Determination of RNA Extraction and Quality

Using the CTAB procedure, we extracted and purified total RNA three times from each of the sample set (Chang et al., 1993). The A260/A280 ratios of all the RNA samples ranged from 1.9 to 2.0. We examined the integrity of all RNA samples by the Agilent 2100 Bioanalyzer, and all the RNA integrity number (RIN) values ranged from 7 to 10.

### cDNA Library Construction and RNA Sequencing

Construction of the cDNA library and RNA sequencing were performed by BIOMARKER (Beijing, China) using the Illumina (San Diego, CA, United States) Genome Analyzer platform in accordance with the manufacturer’s protocols. Paired-end sequencing was performed using a HiSeq 2500 (Illumina) platform with a read length of 125 bp.

### Initial Mapping of Reads

We trimmed reads by removing adapter sequences, reads with too many (> 5%) unknown base calls (N), low-complexity sequences, and low-quality bases (i.e., sequences for which > 65% of the bases had a quality score ≤ 7). HISAT2 (Kim et al., 2015) was used to align all reads of the two species to the *P. euphratica* genome (Ma et al., 2013). Because the intrinsic divergence between the species could result in poor mapping, we did not map RNA-seq reads from the two species onto their own genomes. Next, StringTie (Pertea et al., 2015) created multiple isoforms of genes and estimated the gene expression levels (FPKM) (Trapnell et al., 2010) during assembly. To reduce the effects of background transcription, genes with FPKM ≥ 1 were used for the subsequent analysis. We calculated the Pearson correlation coefficient between biological replicates with R software using the expression data. The Pearson correlation calculated by R was used to evaluate repeatability between biological replicates.

### Analysis of DEGs

We applied Ballgown (Frazee et al., 2015) to determine which transcripts were differentially expressed between two or more experiments, confirming their significance with an *F*-test. Ballgown allows both time-course and fixed-condition differential expression analyses. Therefore, two methods were employed to identify DEGs: (1) time as the main variable and species as the covariate; (2) species as the main variable and time as the covariate.

### Hierarchical Clustering and Gene Co-expression Analysis

Using normalized log_2_ (FPKM+1) values, hierarchical clustering was completed with the pvclust package. Based on the normalized FPKM values, *K*-means clustering was performed by the *K*-Means/K-Medians Support Module (KMS) embedded in MEV 4.9^1^.

### Gene Functional Enrichment and qRT-PCR Analysis

GO and KEGG enrichment analyses of the two differently expressed transcript data sets were performed using a modified Chi-square test and Fisher’s exact test in *R* (*p-*value < 0.01 and false discovery rate < 0.05). Transcription levels of genes were quantified with a MX3005P Real-Time PCR Detection System (Agilent) based on the 2(-delta C(T)) method (Livak and Schmittgen, 2001). The experiment was performed in a 20 μL volume reaction system containing 10 μL 2 × SYBR Premix ExTaq (TaKaRa) with the intercalating dye SYBR Green. All primers were designed using PRIMER5.0 software and are listed in Supplementary Table S4.

## Results

### Physiological and Morphological Changes During Seed Germination

**FIGURE 1:**
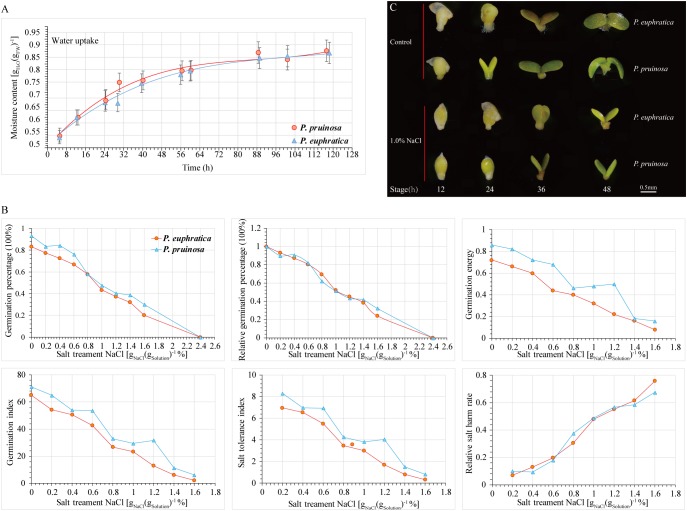
Comparison of physiological indices for *P. euphratica* and *P. pruinosa* seeds under salt stress treatment. **(A)** Time series of changes in moisture content of *P. euphratica* and *P. pruinosa* seeds during germination. **(B)** Effect of different NaCl concentrations on 6 physiological indexes of *P. euphratica* and *P. pruinosa*. **(C)** Morphological changes in *P. euphratica* and *P. pruinosa* seeds from 12 h to 48 h under salt stress and control. Bar = 0.5 mm.

To evaluate the effect of salt stress on seed germination, the progress of seed germination has traditionally been divided into three phases based on seed water uptake during imbibition (Nonogaki et al., 2010). The first phase (phase I) occurs within the period of 0 h–36 h; the plateau phase (phase II) occurs within the period of 36 h–64 h; and phase III is continuous for 64 h–120 h during the transition to seedling growth (Figure 1A). We investigated the relationship between the germination rate and NaCl concentration. Seeds exhibiting high germination rates were selected and cultured in distilled water with a gradient of NaCl concentrations (0%, 0.2%, 0.4%, 0.6%, 0.8%, 1.0%, 1.2%, 1.4%, and 1.6%) (Figure 1B). With increasing NaCl concentration, seed germination was significantly inhibited. At different NaCl concentrations, the seed germination rates of *P. pruinosa* were higher than those of *P. euphratica*. The relative germination percentage of the two species exceeded 80% in the 0.4% NaCl solution, whereas the value approached zero in 2.4% NaCl. We hypothesized that when the relative germination percentages were 75%, 50%, and 25%, the corresponding salt concentrations could be considered suitable, critical and limiting for seed germination, respectively. In our study, for *P. euphratica*, the suitable, critical and limiting values were 0.602%, 1.161%, and 1.72%, respectively, whereas for *P. pruinosa*, these values were 0.599%, 1.179%, and 1.759%, respectively, suggesting that the threshold salinity for the two species differed. We also measured the germination index, salt tolerance index, relative salt harm rate and germination energy (Figure 1B). The average germination percentage, subordinate function values, and threshold salinity for *P. pruinosa* were higher than those for *P. euphratica*. Based on the results, a 1.0% NaCl concentration was selected for the subsequent salt treatment. The seed phenotypes of the two species were observed at four time points (Figure 1C). In the controls, the radicle emergence was completed within 12 h, and the hypocotyl and cotyledons emerged from the seed coat by 24 h. The cotyledons started to open by 36 h and opened fully and turned green by 48 h. In contrast, under the salt treatment, the seeds were still in the imbibition stage at 12 h, the radicle emergence stage was completed by 24 h, and the subsequent stages were all delayed by 12 h.

### RNA-Seq and Mapping of Illumina-Solexa Sequencing Reads

To systematically investigate the transcriptome dynamics of the two species’ seeds during germination under salt stress, we obtained 36 transcriptome samples. After removing low-quality sequences and trimming adapter sequences, 3–6 GB 125-bp paired-end clean reads were generated from each library (Supplementary Table S1). Approximately 80% of the reads matched the genome (Supplementary Table S2). All the genes and transcripts were reassembled (Table 1).

**Table 1:** Numbers of assembled genes and transcriptions.

		0h	4h	12h	24h	48h	72h
*P. euphratica*	Trans Num	68202	73566	79203	69325	78689	81459
	Gene Num	28836	29992	30379	29901	30505	30985
*P. pruinosa*	Trans Num	85952	54047	65547	58952	76926	71959
	Gene Num	30272	26891	29152	27620	30126	29842


In the detection of minor differential gene expression between time points and the two species, we used three biological replicates (Supplementary Figures S1, S2) to assess our data quality. The results showed that the expression values of biological replicates from the same samples were highly correlated (average *R*^2^ > 0.8). Among the genes, FPKM values exceeding 73% ranged from 1 to 100 at each time point (Supplementary Figure S3A). We used the average RPKM of the biological replicates as the expression quantity. To examine the divergence in gene expression between the two species under salt stress in more detail, we performed a hierarchical clustering analysis for all the expressed genes from *P. euphratica* and *P. pruinosa* at each time point using bootstrapping (Supplementary Figure S3B). The correlation dendrogram in Supplementary Figure S3B shows that samples collected at 0, 4 and 12 h clustered together, while those collected at 24, 48 and 72 h clustered into another group. This result indicates that one set of genes was activated during the early stress and germination stages, while there was another set of genes that was differentially expressed after 48 h. Therefore, based on a Spearman correlation analysis, the germinating seed samples from 0, 4 and 12 h were in the early phase, the seeds in the sample from 24 h were in the middle phase, and the seeds from 48 h to 72 h were in the late phase of the germination process.

### Identification of DEGs, Temporal Expression Trends and GO Functional Enrichment

To identify global transcriptional changes that occurred during seed germination under salt stress, we confirmed the two data sets of DEGs, including 12831 DEGs and 19004 differentially expressed transcripts (DETs) for seed germination processes, and 8071 DEGs and 19000 DETs for salt tolerance, of two species. The DEGs were grouped into ten clusters (designated K1–K10) (Supplementary Figure S4) to examine the temporal expression trends of seed germination processes. To better understand the functions of the DEGs and obtain a view of functional transitions across time during seed germination in the two species, GO category enrichment analysis was performed (Supplementary Figure S5) to identify important events (biophysical, biochemical, and cellular processes) during seed germination.

According to the cluster analysis results, all the clusters of *P. pruinosa* and *P. euphratica* could be divided into early (0–12 h), middle (24 h), and late (48–72 h) phases (Supplementary Figure S3B). The early phase (represented by clusters K1 to K4) was strongly expressed at 0–12 h and gradually downregulated between 12 and 72 h in the two species. Based on the GO enrichment results, genes related to “adenyl nucleotide binding,” “adenyl ribonucleotide binding,” “purine ribonucleoside binding,” and “purine nucleoside binding” were increasingly expressed after imbibition (Supplementary Figure S5). Second, some genes associated with “structural molecule activity,” “structural constituent of cytoskeleton,” “intracellular non-membrane-bounded organelle,” “non-membrane-bounded organelle” and “cellular structure restoration” were enriched (Supplementary Figure S5). In addition, some genes associated with “ATP binding” were enriched (Supplementary Figure S5).

Genes in cluster K5 were highly expressed at 0 to 24 h and downregulated from 48 to 72 h. In the middle phase, the enriched genes included genes associated with “catalytic activity,” “mitochondrial part,” “nutrient reservoir activity,” “electron transport chain,” and “respiratory electron transport chain” (Supplementary Figure S5). Each of the five co-expression modules of the two species could be roughly categorized in the late (K6 to K10) phase. Transcripts of these modules were significantly upregulated during at least the last two time points. Many genes of this stage were typified by the enriched functions of “catabolic process,” “generation of precursor metabolites and energy,” “lipid metabolic process,” “carbohydrate metabolic process,” “hydrolase activity,” and “catalytic activity” (Supplementary Figure S5). Moreover, some upregulated genes of this stage were associated with “cellular nitrogen compound biosynthetic process” and “NAD binding” (Supplementary Figure S5).

### Functional Regulatory Network Analysis (KEGG Pathway Enrichment) of Seed Germination Process DEGs

To further elucidate the seed germination process DEGs associated with biochemical pathways, we performed a KEGG pathway enrichment analysis. A total of 3847 out of 12831 DEGs enriched 328 pathways, and 58 pathways were significantly (*p-*value ≤ 0.01) overrepresented during seed germination (Supplementary Figure S6).

The early phase was exemplified by an observed statistically significant enrichment of “ribosome,” “proteasome,” and “protein processing in endoplasmic reticulum” pathways (Supplementary Figure S6). The middle phase exhibited the enrichment of “flavonoid biosynthesis,” “oxidative phosphorylation,” “ribosome,” “proteasome” and “spliceosome” pathways (Supplementary Figure S6). While many genes related to the metabolism of free amino acids were enriched in phase III (Supplementary Figure S6), most of the major pathways were enriched in the late phase, including “carbon metabolism,” “glycolysis/gluconeogenesis,” “starch and sucrose metabolism,” “oxidative phosphorylation,” “photosynthesis,” “porphyrin and chlorophyll metabolism,” and “carotenoid biosynthesis” (Supplementary Figure S6). “Oxidative phosphorylation” provides ATP for other metabolism pathways, such as mitochondrial repair and differentiation (Weitbrecht et al., 2011). The glyoxylate pathway contains a key step in the conversion of fatty acids to sucrose (Pritchard et al., 2002).

### DEGs Related to Energy Production for Seed Germination Processes

**FIGURE 2:**
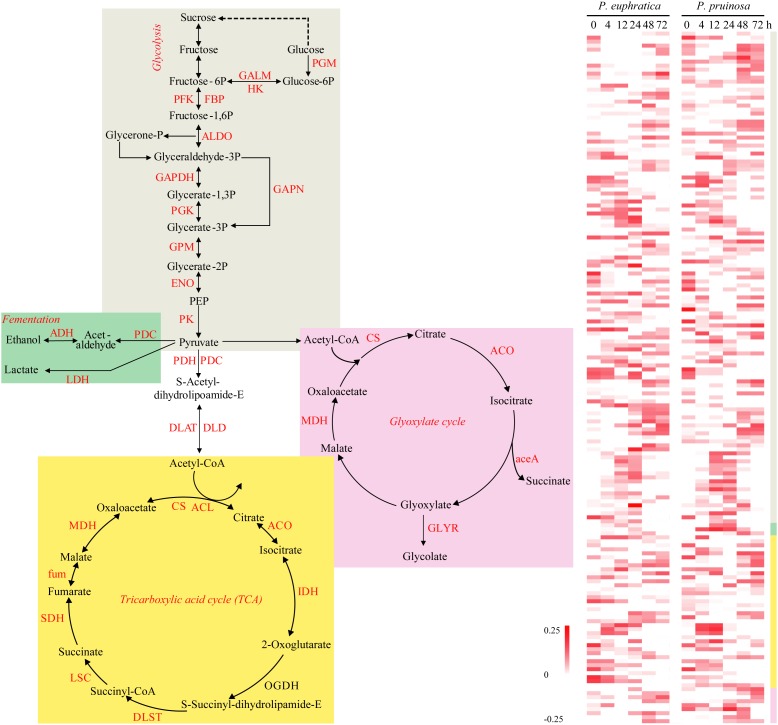
The energy production during seed germination and its possible relationship to early transcriptome changes in *P. euphratica* and *P. pruinosa*. Glycolysis (gray region), TCA cycle (yellow region) and fermentation respiration (green region) are the common pathways associated with ATP production. Glyoxylate (pink region) is also an important energy source for the germinating seed via lipid metabolism. The colors behind the heat map represent expression patterns of the counterpart genes in the four pathways. For details of abbreviations, see Supplementary Table S3.

During the preliminary phase, due to the inactivation of photosynthesis, the degradation of storage needed for energy production via processes such as glycolysis, the glyoxylate cycle, and the tricarboxylic acid (TCA) cycle, largely determines germination vigor. We defined the relative functional categories to be “carbon metabolism,” “glyoxylate and dicarboxylate metabolism,” “glycolysis/gluconeogenesis,” and “starch and sucrose metabolism.” Then, we identified the four major energy production processes, i.e., fermentation, the TCA cycle, glyoxylate and glycolysis, representing significantly overrepresented functional pathways, and we examined the expression patterns of the related DEGs (Figure 2). Here, ten gene families participating in the TCA cycle were differentially expressed over time in the two species. With respect to glycolysis, numerous gene families were upregulated, such as GALM, PFK, FBP, ALDO, GAPDH, and PK. In anaerobic respiration, three related gene families, PDC, ADH, and LDH, were all upregulated in the two species.

### Hormonal Regulation of Seed Germination in *P. euphratica* and *P. pruinosa* Under Salt Stress

**FIGURE 3:**
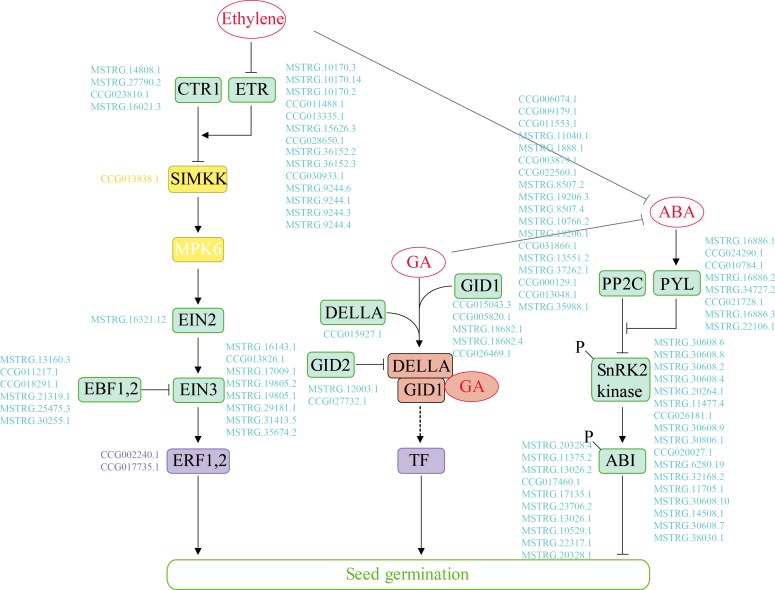
The Ethylene, GA and ABA major metabolic pathways in the germinating seeds of *P. euphratica* and *P. pruinosa* under salt stress. Promotion or inhibition is indicated by thick arrows and blocks, respectively. Enzymes and genes are shown. A MAPK cascade (yellow box) consisted of SIMKK and MPK6. Examples of transcription factors (purple box) and other regulators (green box) contributing to these processes are presented. Genes are indicated with blue letters. The GA-GID1-DELLA complex (red box) is recognized by an F-box protein (GID2) for ubiquitination in the SCF complex (included in the dotted arrow). For details of abbreviations, see Supplementary Table S3.

In our study, 100 genes associated with “plant hormone signal transduction” were differentially expressed over time in the two species. We identified the key hormone signal transduction genes and further compared the expression profiles of the multi-step signaling pathways of ABA, GA and ethylene (Figure 3 and Supplementary Figure S7). Genes related to ABA signal transduction, e.g., PYL/PYR1, the negative regulator PP2C and the positive regulator SnRK2, exhibited similar expression patterns in the two poplars. The expression level of PP2C was high at 0 and 4 h but decreased after 12 h. In the GA signaling pathway, DEGs exhibited different regulatory expression patterns between the species during germination under salt stress. Specifically, the DELLA protein expression was upregulated from 0 to 12 h in *P. euphratica* but was continuously high level in *P. pruinosa*. GID1 was strongly upregulated during the middle and late phases of seed germination, while the expression of specific genes differed between the two species. Furthermore, most GA signal transcription-related genes were upregulated in the middle and late phases. We also identified the genes involved in ethylene signaling, as shown Figure 3. The expression pattern analysis indicated that most of the DEGs exhibited similar expression patterns in the two species for ETR and EIN3 (Supplementary Figure S7). CTR expression was upregulated in the late phase, while ETR was highly expressed after the early phase of germination in the two species.

### Transcription Factors and Genes Involved in Salt Responses During Seed Germination

**FIGURE 4:**
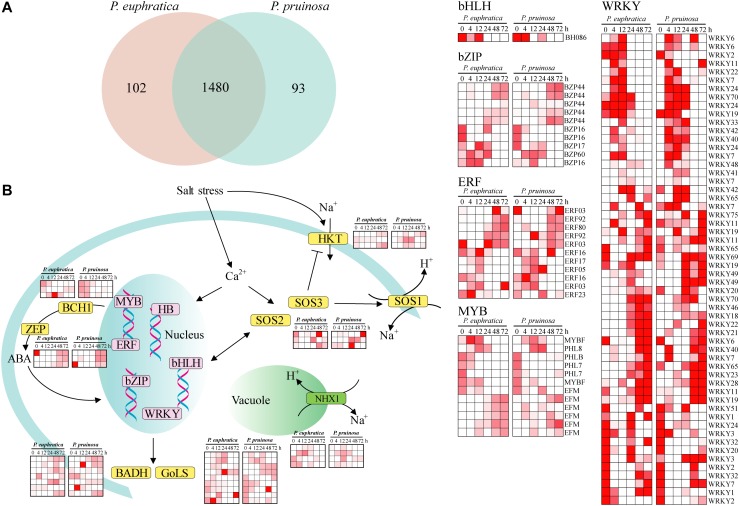
The current known components and relationships of transcription factors related to salt response in *P. euphratica* and *P. pruinosa.* **(A)** Venn diagram showing overlaps between the transcription factors of *P. euphratica* and *P. pruinosa*. **(B)** The expanded and positively selected genes in the salt response pathways of *P. euphratica* and *P. pruinosa* (yellow). Expression of the differently expressed transcription factors of *P. euphratica* and *P. pruinosa* (pink). The heatmap was generated from hierarchical cluster analysis of genes.

Numerous transcription factors that regulate the response to salt stress in desert poplars have been identified (Trapnell et al., 2010). Here, a total of 1582 and 1573 expressed transcripts were categorized as transcription factors in *P. euphratica* and *P. pruinosa*, respectively (Figure 4A). In total, 1480 transcription factors were expressed in both species (Figure 4B). Relatively few genes displayed species-specific expression. *MYBs*, *bZIPs*, *WRKY*, and *ERF*, as key response factors to abiotic stresses, were all induced by salt stress (Figure 4B), and the changes in their expression dynamics may reveal their critical functions in response to salt stress (Yamaguchi-Shinozaki and Shinozaki, 2005). Furthermore, some proteins regulating Na^+^/H^+^ transport and controlling ion homeostasis, such as NHXs, SOS1, SOS2, SOS3, and HKTs, were induced by salt stress (Figure 4). These results confirm that the genes related to ion transport and chloride channels play vital roles in maintaining and re-establishing homeostasis in the cytoplasm (Hasegawa et al., 2000; Wang et al., 2008; Sun et al., 2009; Ye et al., 2009; Qiu et al., 2011). *BCH1* and *ZEP*, which are involved in the biosynthesis of ABA, were highly upregulated in salt-stressed samples in the two species (Figure 4B). In addition, the expression of BADH and GolS, which are involved in critical solute biosynthesis processes that help plants maintain high osmotic pressure under salt stress. (Taji et al., 2002; Bartels and Sunkar, 2005), was induced by the salt treatment. Nevertheless, the expression patterns of genes responding to salt stress in *P. euphratica* were consistent with those in *P. pruinosa*, indicating there is extensive transcriptional consistency in the two species with respect to their responses to salt stress.

### GO Functional Enrichment Between the Two Species Over the Time Series

The temporal expression trends of DEGs between the two species during germination were obviously different, suggesting that the two desert poplars might have evolved different gene expression patterns to adapt to different salty desert habitats. To obtain a better view of the functional differences between the species over the course of germination, GO enrichment analysis was employed, comparing the two species in two phases (the middle phase had only one DEG) (Supplementary Figure S8). The results indicated that in the early phase, 2766 DEGs were mainly enriched, and these DEGs were associated with the functional classifications “ribosomes,” “amide biosynthetic process,” “cellular macromolecule biosynthetic process,” “protein activity,” and “ATP binding.” In the late phase, 5305 enriched DEGs were associated with “response to stress,” “response to oxidative stress,” “response to abiotic stress,” “response to stimulus,” “ATP metabolic process,” “photosystem,” “photosynthesis,” “growth,” “developmental process,” “ion binding,” “calcium ion binding,” and “oxidoreductase activity.”

### KEGG Functional Enrichment in the Two Species Over the Time Series

**FIGURE 5:**
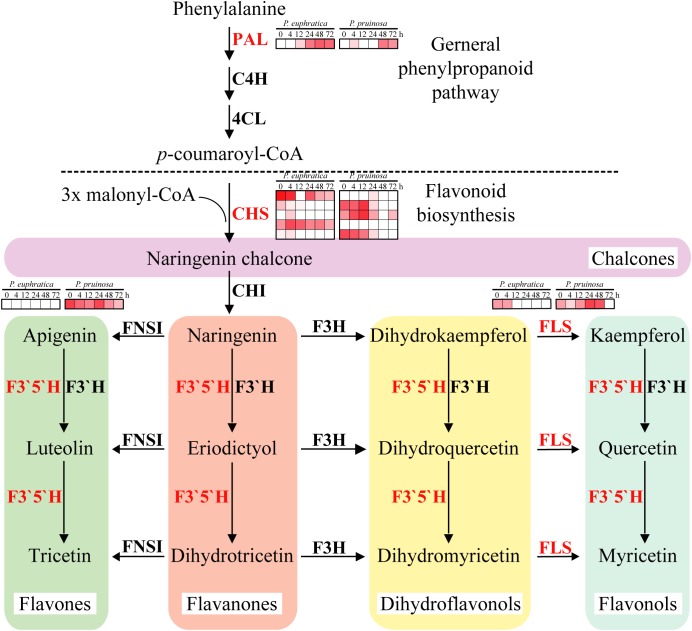
Regulatory network of flavonoid biosynthesis underlying the co-regulated DEGs in *P. euphratica* and *P. pruinosa*. For details of abbreviations, see Supplementary Table S3.

To further elucidate the different enriched biochemical pathways, DEGs of the two species were mapped into 352 pathways, 11 of which were significantly (*p-*value ≤ 0.01) enriched, including “flavonoid biosynthesis,” “stilbenoid, diarylheptanoid and gingerol biosynthesis,” “brassinosteroid biosynthesis,” “phenylpropanoid biosynthesis,” “diterpenoid biosynthesis,” and “monoterpenoid biosynthesis” (Supplementary Figure S9). The results indicate that many antioxidants, antioxidases and secondary metabolites are involved in the adaptation to salt stresses by these two species (Burritt and Mackenzie, 2003). The second metabolite in the flavonoid pathway plays vital roles in stress protection, but the biosynthesis of this metabolite is regulated by key enzymes (Winkel-Shirley, 2002). In this study, *PAL* was induced at 12 h in *P. euphratica* seeds and at 48 h in *P. pruinosa* seeds (Figure 5). CHS, whose five gene copies had different expression patterns between the two species, initiated flavonoid biosynthesis. Furthermore, the FLS expression in *P. pruinosa* was higher than that in *P. euphratica*. Specifically, FLS was highly expressed in the early phase in *P. euphratica* and was significantly and highly expressed during the seed germination process. In addition, the expression levels of F3′5′H and CHS in *P. pruinosa* were significantly higher than those in *P. euphratica* (Figure 5). F3H converts naringenin to dihydrokaempferol which is further converted to kaempferol and quercetin by FLS. The duplication of FLS may allow the ability to diversify the types and amounts of flavonols produced in different tissues and under different stresses(Winkel-Shirley, 2002).

**FIGURE 6:**
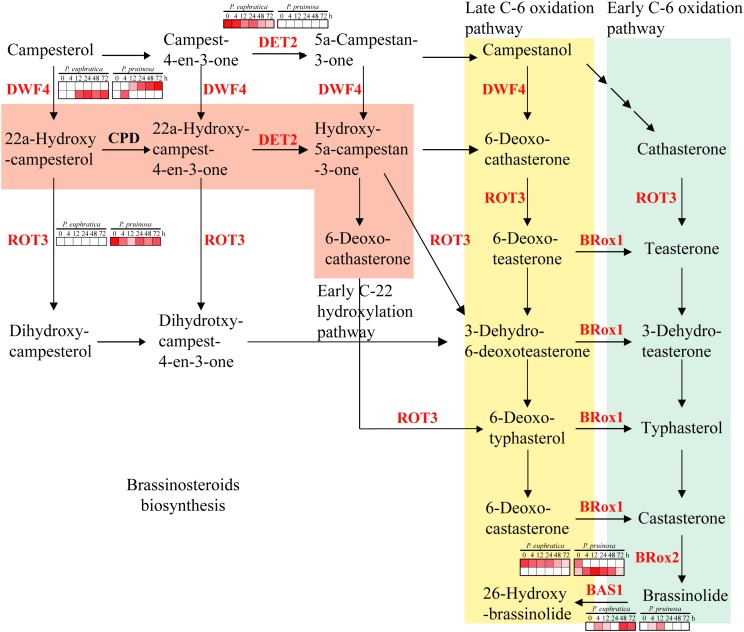
Regulatory network of brassinosteroid biosynthesis underlying the co-regulated DEGs in *P. euphratica* and *P. pruinosa*. For details of abbreviations, see Supplementary Table S3.

Brassinosteroids are involved in an extensive range of effects, such as cell division, cell expansion, xylem differentiation and seed germination, in plants (Kagale et al., 2007). In the two species, six gene families related to brassinosteroid metabolism were enriched, including DET2, DWF4, BR6OX1, BRox2, ROT3 and BAS1. Among them, *DET2* and *BAS1* were highly expressed in *P. euphratica* and exhibited relatively low expression in *P. pruinosa*, while *ROT3* was highly expressed in *P. pruinosa* but was not detected in *P. euphratica* (Figure 6). Moreover, there were two copies of both *DWF4* and *BR6OX2*, and each copy exhibited a different expression pattern between the two poplars.

### ROS Level and Enzyme Activity Determination

**FIGURE 7:**
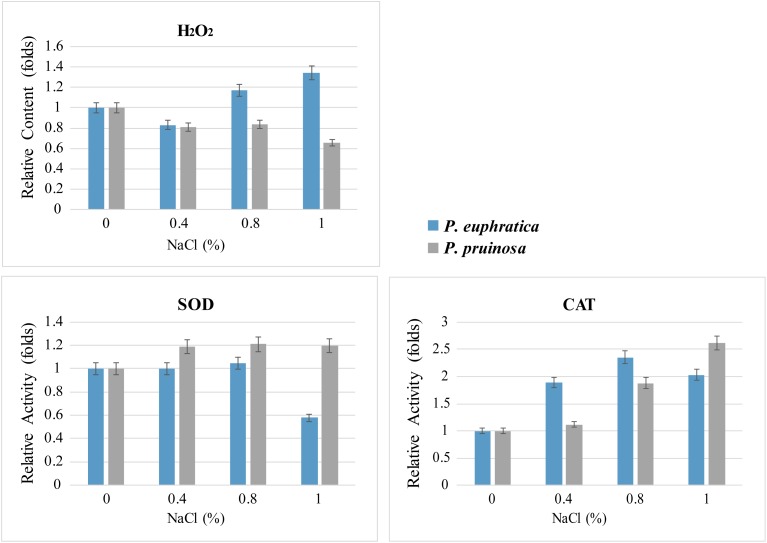
Quantitative comparison of superoxide contents and antioxidant enzyme activities (SOD and CAT) for the two species exposed to salt stress. The changes in content and activity were analyzed with different salt treatments.

We measured ROS levels and related enzyme activities. The quantification assay indicated that more hydrogen peroxide accumulated in *P. euphratica* than in *P. pruinosa* under the various salt conditions, especially in 1.0% NaCl, where the levels were approximately 2-fold higher in *P. euphratica* than in *P. pruinosa* (Figure 7). Therefore, SOD activities were significantly higher in *P. pruinosa* than in *P. euphratica* after treatment with 0.4% NaCl solution. The CAT activities in the treatment with 1.0% NaCl solution demonstrated a similar pattern.

### Verification of Expression Patterns by qRT-PCR

**FIGURE 8:**
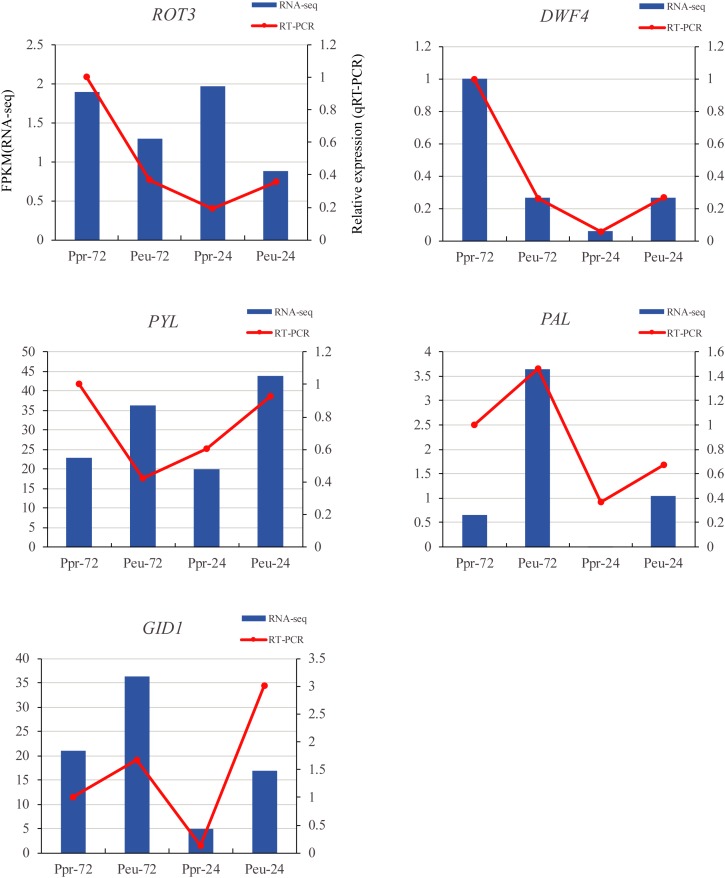
qRT-PCR verification of five selected DEGs. We compared the RNA-seq data (blue bar) with qRT-PCR data (red lines). And we indicated the normalized expression level (FPKM) of RNA-seq on the y-axis to the left. The relative qRT-PCR expression level is shown on the y-axis to the right. *CYC063* was set as the internal control. Both methods agree, showing similar gene expression trends.

To validate the RNA-seq results, qRT-PCR analysis was conducted at different time points during seed germination in the two species (Figure 8). The results of genes studied by the RT-PCR analysis, including those in the plant hormone signal transduction (*PYL* and *GID1*), flavonoid biosynthesis (*PAL*) and brassinosteroid biosynthesis (*ROT3* and *DWF4*) pathways, were all similar to the RNA-seq results.

## Discussion

### *P. pruinosa* Showed a Higher Salt Tolerance Than *P. euphratica* at the Three Seed Germination Stages

*Populus euphratica* and *P. pruinosa* diverged from a recent common ancestor between 1 and 2 million years ago (Wang J. et al., 2011) and exhibited different ecological adaptations to desert habitats. In this context, the two desert poplars have evolved different genetic strategies (Ma et al., 2013; Zhang et al., 2013). However, it is not known whether these genetic variations also underlie differences in seed germination.

In the present study, the rate of seed germination in *P. pruinosa* faster than that in *P. euphratica* during seed germination (Figure 1C). Based on the seed moisture content, the seed germination time courses for the two species, upon the transfer of seeds to water, can be divided into three phases, which agree with the three classical phases of seed germination (Nonogaki et al., 2010; Figure 1A). We also investigated the relationship between the germination rate and NaCl concentration. The average germination percentage, subordinate function values, and threshold salinity of *P. pruinosa* were higher than those of *P. euphratica*. Based on transcriptome analysis, approximately 80% of the reads matched with the genome, and the number of mapped genes in each library was 52%–70%, indicating that most genes were expressed in the seeds of the two species under salt stress. The correlation dendrogram is consistent with the separate phases classified by seed water uptake (Supplementary Figure S3B), suggesting that, in two stages (early and late phases), the sister species evolved divergent regulatory and metabolic pathways associated with seed germination in different salt habitats.

### Biochemical Processes of Poplar Seeds Are Regulated by Highly Coordinated Transcript Dynamics

The expression data in the three seed germination phases showed a high reproducibility in both species, and each phase was clearly distinguished by expression dynamics. The DEGs induced early in seed germination (early phase) appeared to be associated with the repair of genetic materials, the cellular structure and the resumption of energy metabolism. During seed germination, the free amino acids involved in protein synthesis are provided by storage protein degradation induced by osmopriming in the first hours of imbibition (Wang et al., 2012). Accordingly, proteases are newly synthesized and accumulate during imbibition (Yang et al., 2007). Therefore, we speculate that in *P. euphratica* and *P. pruinosa*, amino acid biosynthesis genes are expressed after 48 h of the seed germination process, and their products provide for the synthesis and metabolism of *de novo* proteins in the growing embryo (Joosen et al., 2013). Thus, the stored proteins in seeds act not only as important sources of amino acids but also as a source of energy (Angelovici et al., 2011). The middle phase was associated with the active nutrient reservoir, amino acid metabolism and catalytic activity. In this stage, producing a redox state is likely a primary function of the fast recovery of cellular metabolism at the beginning of imbibition (Rosental et al., 2014). The functions of the enriched genes not only produce energy but also promote the activity of essential enzymes to support the completion of germination (Van Dongen et al., 2011). Moreover, flavonoids can induce a delay in the germination rate and play important roles in protection against diverse stresses (D’Auria and Gershenzon, 2005). Most flavonoid genes in this study were enhanced in *P. pruinosa* in the middle phase, indicating that the “flavonoid biosynthesis” pathway might lead to a difference in the seed germination rate between *P. pruinosa* and *P. euphratica*.

Reserves used for the germination of seeds are primarily stored in the form of starch, lipids and proteins in the embryo or endosperm (Yang et al., 2009). Proteins related to hydrolase activity contribute to starch and protein degradation (Rosental et al., 2014), while catalytic activity proteins may increase enzyme activities or provide the energy required during seed germination. Nitrogen-containing compounds release seeds from dormancy, presumably leading to the oxidation of NADPH and therefore providing an increased carbon flow through the glycolytic and oxidative pentose phosphate pathways (PPP) (Roberts, 1964; Hendricks and Taylorson, 1974; Roberts and Lord, 1979; Cohn et al., 1983; Hilhorst and Karssen, 1989). NADP, as a coenzyme of glucose-6-phosphate dehydrogenase, plays a key role in linking the glycolysis pathway and PPP (Nonogaki et al., 2010). Here, we investigated the DEGs involved in fermentation, the TCA cycle, glyoxylate and glycolysis during seed germination. We found that energy production mainly occurred in the later phase and increased gradually. Interestingly, many genes of key enzymes in the TCA cycle were expressed during the early phase, which would lead to the accumulation of many key enzymes during early germination (Weitbrecht et al., 2011). In the late phase, some of the enriched genes were linked to “the photosystem II oxygen evolving complex,” “photosystem,” and “photosynthesis,” suggesting that the seeds had already started to photosynthesize, contributing to the energy supply and powering the productivity of the seed (Ruuska et al., 2004; Goffman et al., 2005; Allen et al., 2009). Meanwhile, “glyoxylate metabolism” activity was enriched in the last phase of germination, which suggests that lipid metabolism is also an important energy source for seed germination. The results demonstrated that activation of energy metabolism during early germination is necessary for seed germination, however, energy production is more complex in the late phase than in earlier phases. In addition to energy metabolism, the expression of genes associated with detoxification were also involved in responses to salt stress in the two desert poplars (Zhu, 2001); these genes included genes associated with “glutathione metabolism,” “flavonoid biosynthesis” and “cytochrome P450,” which all are tightly correlated with seed tolerance to salt stress (Alscher, 1989). These observations suggest that *P. euphratica* and *P. pruinosa* quickly establish energetic and developmental balances under salt stress. Germination is actuated by a large number of cellular processes, such as transcription, translation, repair mechanisms, responses to various stresses, organelle reassembly and cellular structure reconstruction. All the processes are supported by metabolism for energy generation. Together, the above results indicate that the transition of primary biochemical processes over time during the seed germination of the two studied poplars is produced partly by highly coordinated transcript dynamics. As the two desert poplars have adapted to different salty desert habitats, these species may have developed different genetic pathways under salt stress during seed germination.

### Hormonal Regulation Contributes to the Difference in Seed Germination Phases

Some genes were enriched in the “plant hormone signal transduction” functional category, which is key for physiological state determination and the regulation of seed germination, especially the GA-ABA balance (Meyer et al., 2009). ABA positively regulates the induction of dormancy and negatively regulates germination. Here, the genes related to ABA signal transduction exhibited similar expression patterns in the two poplars. For example, *PYL*/*PYR1*, which are considered ABA receptors, exhibited upregulated expression during the first stage, suggesting that the ABA content of the dry seeds was high and decreased during imbibition (Preston et al., 2009). In addition, the negative regulator PP2C has been found to be a major core component of ABA signaling; its expression level was high at 0 and 4 h but decreased after 12 h (Fujii and Zhu, 2009; Umezawa et al., 2009).

GAs play an important role in the promotion of germination and the release of dormancy (Kucera et al., 2005) by stimulating ABA degradation. Here, many DEGs associated with the GA signaling pathway exhibited different expression patterns during germination under salt stress. Specifically, DELLA proteins belonging to the GRAS family were negatively regulated in the GA signaling pathway (Sun and Gubler, 2004) and upregulated from 0 to 12 h in *P. euphratica*, while they were continuously expressed at high levels in *P. pruinosa*. *GID1*, coding a soluble GA receptor, was strongly upregulated during the middle and late phases of seed germination. The GA protein can interact with DELLA when bioactive GAs are present (Ueguchi-Tanaka et al., 2007). Furthermore, most GA signaling transcription-related genes were upregulated in the middle and late phases, which corresponds to the results of a previous study showing that the GA content increased during germination in seeds during phase II.

Ethylene is implicated in the promotion of germination in many species. Here, we identified the DEGs involved in ethylene signaling in seed germination. We found that most of these DEGs, alongside *ETR* and *EIN3*, exhibited similar expression patterns in the two species (Supplementary Figure S7). In the absence of ethylene, ETR1 activates CTR1, which negatively regulates downstream signaling components and is inactive in the presence of ethylene. *CTR* expression was upregulated in the late phase of germination in the two species, while ETR was highly expressed after the early phase of germination. These proteins are regulated by ethylene levels during seed germination by the inactivation of a MAPK cascade comprising SIMKK and MPK6, which are positive regulators of the ethylene response pathway (Ouaked et al., 2003). EIN3 and EIN3-LIKE proteins bind to the promoter of the *ERF1* (ethylene responsive factor 1) gene and thereby confer a hierarchy of transcription factors involved in ethylene signaling (Lee and Kim, 2003). Most importantly, the expression patterns of DEGs related to the ethylene pathway were different between *P. euphratica* and *P. pruinosa*, indicating that ETR and EIN3 distinctly regulate ethylene signal transcription pathways during seed germination.

Overall, GAs increase and counteract ABA inhibition in the early and late phases of germination (North et al., 2010). Ethylene counteracts ABA inhibition by interfering with ABA signaling during the late phase of germination, while the ABA content is regulated by an equilibrium between the biosynthesis and catabolism of ABA (Nambara and Marion-Poll, 2005). Thus, many of the DEGs exhibited analogous expression patterns in the two species in the models for GA, ABA and ethylene in response to salinity stress but exhibited completely different expression patterns during seed germination.

### The Fine Regulation of the Synthesis of Flavonoids and Brassinosteroids in Desert Poplars Contributes to Their Environmental Adaptation

Flavonoids have an extensive range of biological functions, including protecting plants under various stresses (Winkel-Shirley, 2002). Flavonoids are synthesized by the phenylpropanoid pathway and found in most seeds and grains; the major types of flavonoids in seeds are flavonols, anthocyanins, phlobaphenes, isoflavones and proanthocyanidins (Lepiniec et al., 2006). Several genes that encode key enzymes in the flavonoid biosynthetic pathway were expressed differently between the seeds of *P. euphratica* and *P. pruinosa* under salt stress (Figure 5). We suggest that the phenylpropanoid pathway, especially the flavonoid metabolism pathway, is widely involved in protection from salt stress in both desert poplars. In general, salt stress is often accompanied by an oxidative burst in plants. In this study, the hydrogen peroxide (H_2_O_2_) accumulation in *P. euphratica* was 2-fold higher than that in *P. pruinosa* under the various salt conditions, especially in 1.0% NaCl (Figure 7), suggesting that salt treatment might induce oxidative stress in the seeds of *P. euphratica*. The unavoidable accumulation of H_2_O_2_ and scavenging pathways activity should be maintained in balance, where H_2_O_2_ could either perform a signaling role or reach a nontoxic level in plants under salt stress conditions. To alleviate and eliminate highly reactive oxygen species, plants have evolved a battery of antioxidative mechanisms, and the antioxidant defense system includes hydrophilic and hydrophobic antioxidants and enzymes such as SOD and CAT (Shalata and Tal, 1998). SOD activities in *P. pruinosa* were significantly higher than those in *P. euphratica* when the seeds were exposed to concentrations of NaCl above 0.4%, while CAT activities in *P. pruinosa* were also higher than those in *P. pruinosa* when seeds were treated with 1.0% NaCl. Both antioxidases could play a crucial role in scavenging redundant ROS (H_2_O_2_) induced by salt stress. Altogether, a significant proportion of the antioxidants induced by salt stress were secondary metabolites, such as a vast amount of compounds primarily derived by the phenylpropanoid pathway (Dixon and Paiva, 1995).

Brassinosteroids are involved in a wide range of growth and development aspects in plants (Kagale et al., 2007). One of the most interesting influences of brassinosteroids is their ability to confer resistance to various abiotic stresses. Several brassinosteroid biosynthesis genes have been identified by molecular genetic analysis and reverse genetic analysis (Takahashi et al., 2005). Among the gene families enriched in the brassinosteroid pathway, *DET2* and *BAS1* were highly expressed in *P. euphratica* and exhibited relatively low expression in *P. pruinosa*, while *ROT3* was highly expressed in *P. pruinosa* but was not detected in *P. euphratica* (Figure 6, 8). Moreover, *DWF4* and *BR6OX2* each contain two copies, and each copy exhibited a different expression pattern between the two poplars. The results suggest that the fine regulation of the synthesis of brassinosteroids in desert poplars contributes to their environmental adaptation.

## Conclusion

In this study, a multidimensional transcriptome dataset allowed us to discern highly dynamic and coordinated gene expression, as well as functional and regulatory shifts exhibited by the germinating seeds of two species in response to continuous salinity stress. Based on these results, we conclude that the fine regulation of the synthesis of flavonoids and brassinosteroids in desert poplars contributes to their environmental adaptation.

## Data Availability

The Illumina sequencing data sets are available at the NCBI Sequence Read Archive (SRA) database with the project accession number: PRJNA484685.

## Author Contributions

DW conceived and designed the experiments. CZ, WL, and YL conducted the bioinformatic work and wrote the manuscript. XuZ, XB, and ZN contributed to conducting experiments for physiology and transcript analysis. XiZ and ZL provided assistance in sample collection. All authors read, revised and approved the final manuscript.

## Conflict of Interest Statement

The authors declare that the research was conducted in the absence of any commercial or financial relationships that could be construed as a potential conflict of interest.

## Footnotes

1 http://www.tm4.org/mev

## Supplementary Material

FIGURES S1, S2 Reproducibility of each trio of biological replicates. The samples were collected at different time points, and total RNA isolation was used to construct RNA-seq libraries for them independently. FPKM values of all the genes expressed in at least one of the 36 sequenced samples are shown in scatter plots and were used as input for the Pearson product-moment correlation coefficient analysis. The correlations between the biological replicates were high in both two species [average *r* = 0.945 in *P. euphratica* (Supplementary Figure S1) and *r* = 0.939 in *P. pruinosa* (Supplementary Figure S2)].

FIGURE S3 Number of genes expressed at each time point **(A)** and hierarchical clustering of six time points for *P. euphratica* and *P. pruinosa* **(B)**.

FIGURE S4 Hierarchical clustering of six time points for *P. euphratica* and *P. pruinosa*. A to B, the expression patterns of co-expression modules of *P. euphratica* **(A)** and *P. pruinosa* **(B)**, ordered according to the sample time points of their peak expression. **(C)** The gene numbers and the expression fitted curves of all the modules in A and B. For each gene, the FPKM value normalized by the maximum value of all FPKM values of the gene over all time points is shown.

FIGURE S5 GO function enrichment of the DEGs for seed germination processes.

FIGURE S6 KEGG function enrichment of the DEGs for seed germination processes.

FIGURE S7 The expression pattern of the hormone-related genes in the two poplars. Expression patterns of hormone-related genes. Normalized expression levels of genes related to ethylene, GA and ABA are shown.

FIGURE S8 GO function enrichment of the DEGs for salt tolerance variety of the two species.

FIGURE S9 KEGG function enrichment of the DEGs for salt tolerance variety of the two species.

TABLE S1 Overview of the data size of all the samples of *P. euphratica* and *P. pruinosa*.

TABLE S2 Summary of the illumine sequencing reads and the matches in the *P. euphratica* and *P. pruinosa*.

TABLE S3 The details of abbreviations.

TABLE S4 Primer used for real-time quantitative PCR in this study.
