# Supplementary figures and images for: Transcriptomic Analysis of Seed Germination Under Salt Stress in Two Desert Sister Species (Populus euphratica and P. pruinosa)
Source: Front Genet. 2019 Mar 25;10:231. doi: 10.3389/fgene.2019.00231 (PMC6442517; doi:10.3389/fgene.2019.00231)

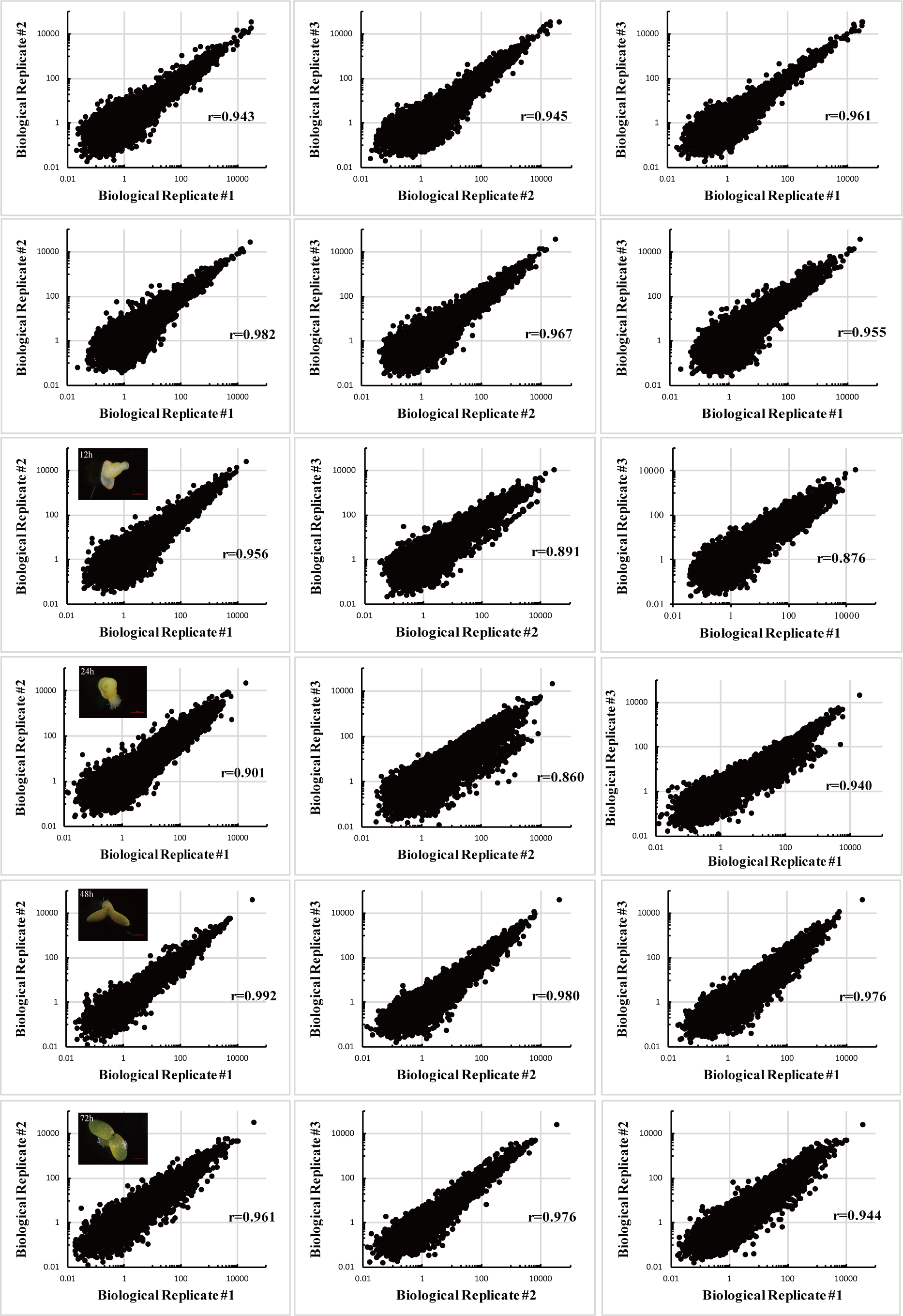

Supplement: FIGURES S1, S2 — Reproducibility of each trio of biological replicates. The samples were collected at different time points, and total RNA isolation was used to construct RNA-seq libraries for them independently. FPKM values of all the genes expressed in at least one of the 36 sequenced samples are shown in scatter plots and were used as input for the Pearson product-moment correlation coefficient analysis. The correlations between the biological replicates were high in both two species [average r = 0.945 in P. euphratica (Supplementary Figure S1) and r = 0.939 in P. pruinosa (Supplementary Figure S2)]. [file Image_1.TIF]

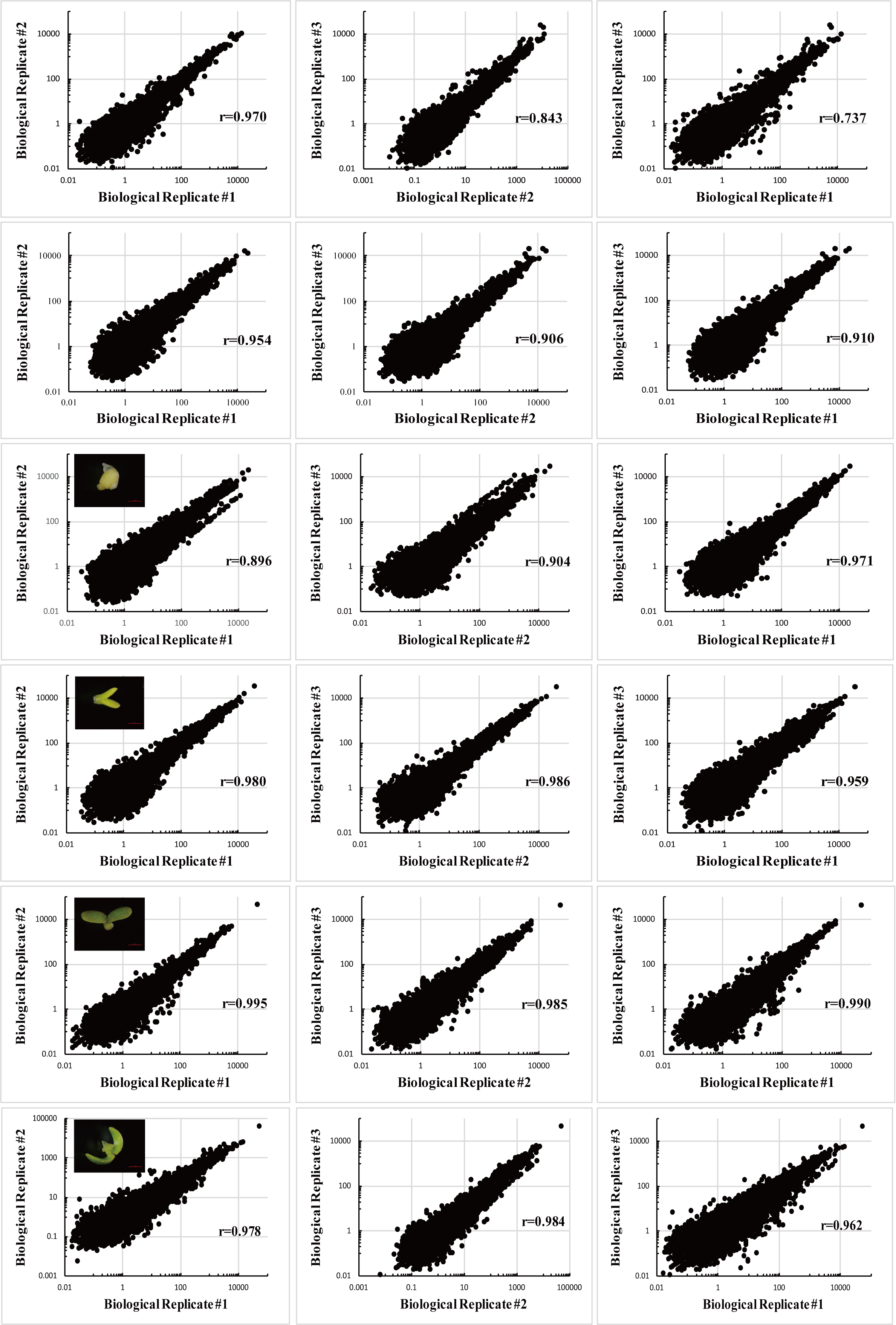

Supplement: Supplementary file 2 [file Image_2.TIF]

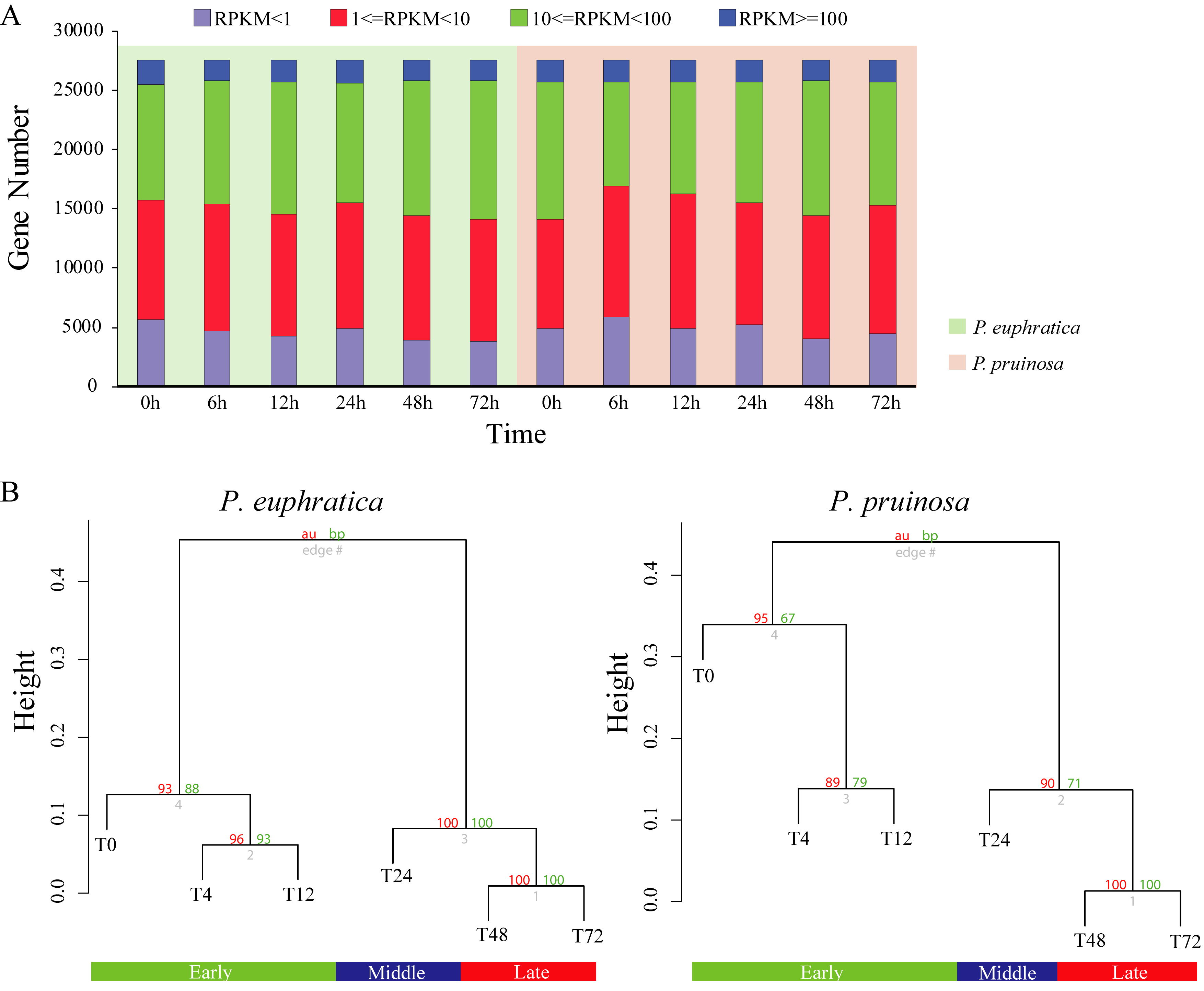

Supplement: FIGURE S3 — Number of genes expressed at each time point (A) and hierarchical clustering of six time points for P. euphratica and P. pruinosa (B). [file Image_3.TIF]

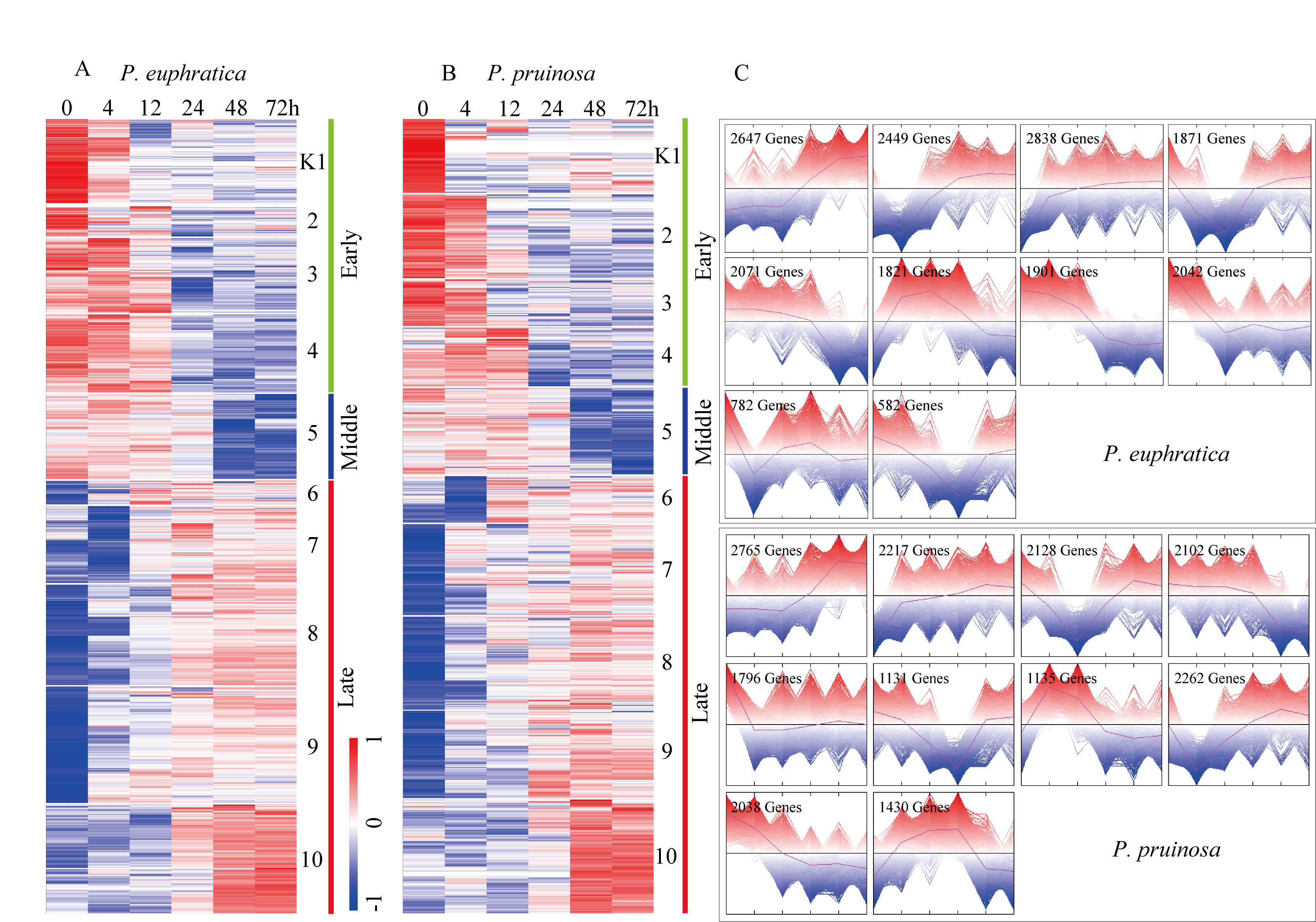

Supplement: FIGURE S4 — Hierarchical clustering of six time points for P. euphratica and P. pruinosa. A to B, the expression patterns of co-expression modules of P. euphratica (A) and P. pruinosa (B), ordered according to the sample time points of their peak expression. (C) The gene numbers and the expression fitted curves of all the modules in A and B. For each gene, the FPKM value normalized by the maximum value of all FPKM values of the gene over all time points is shown. [file Image_4.TIF]

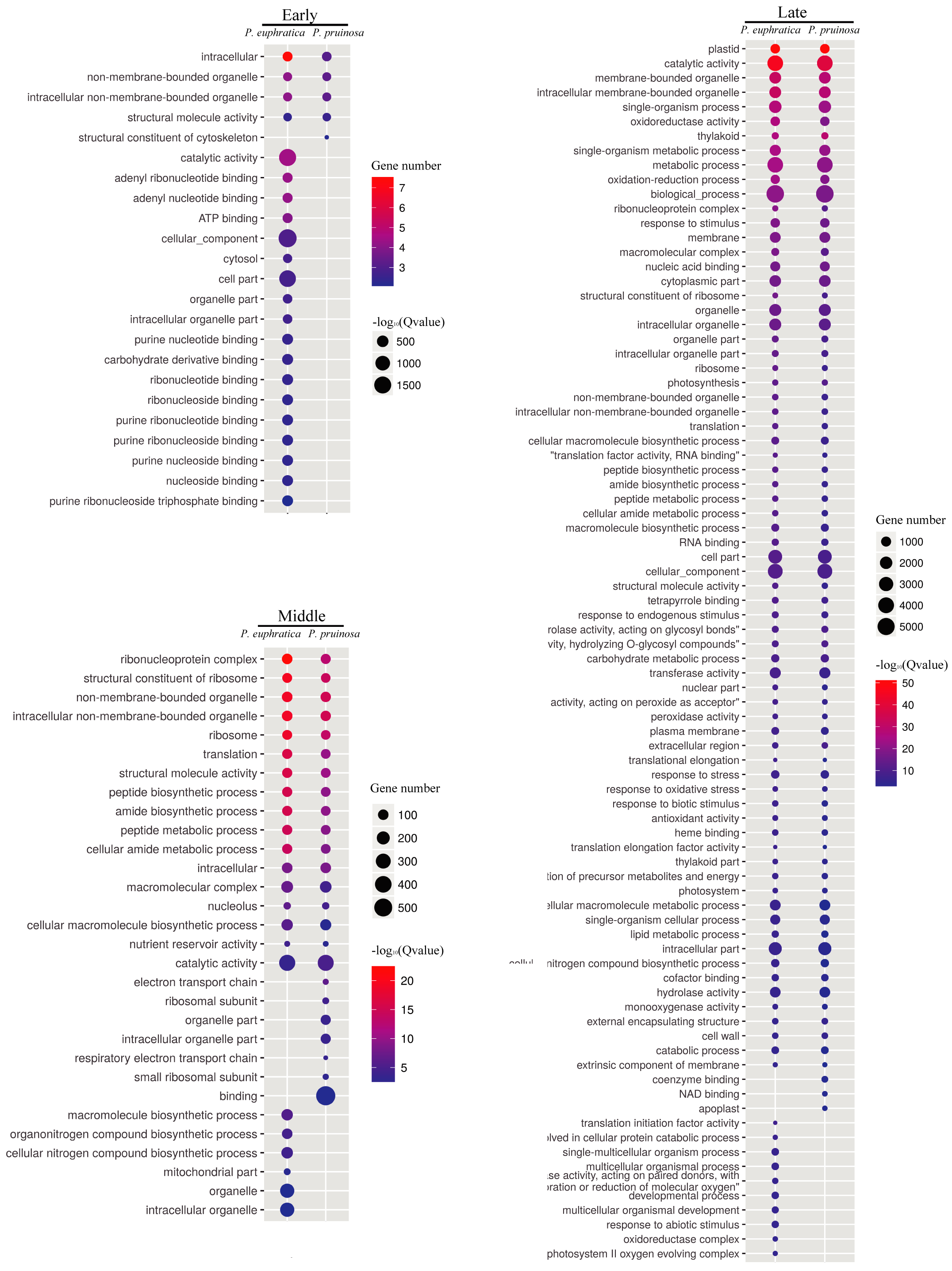

Supplement: FIGURE S5 — GO function enrichment of the DEGs for seed germination processes. [file Image_5.tif]

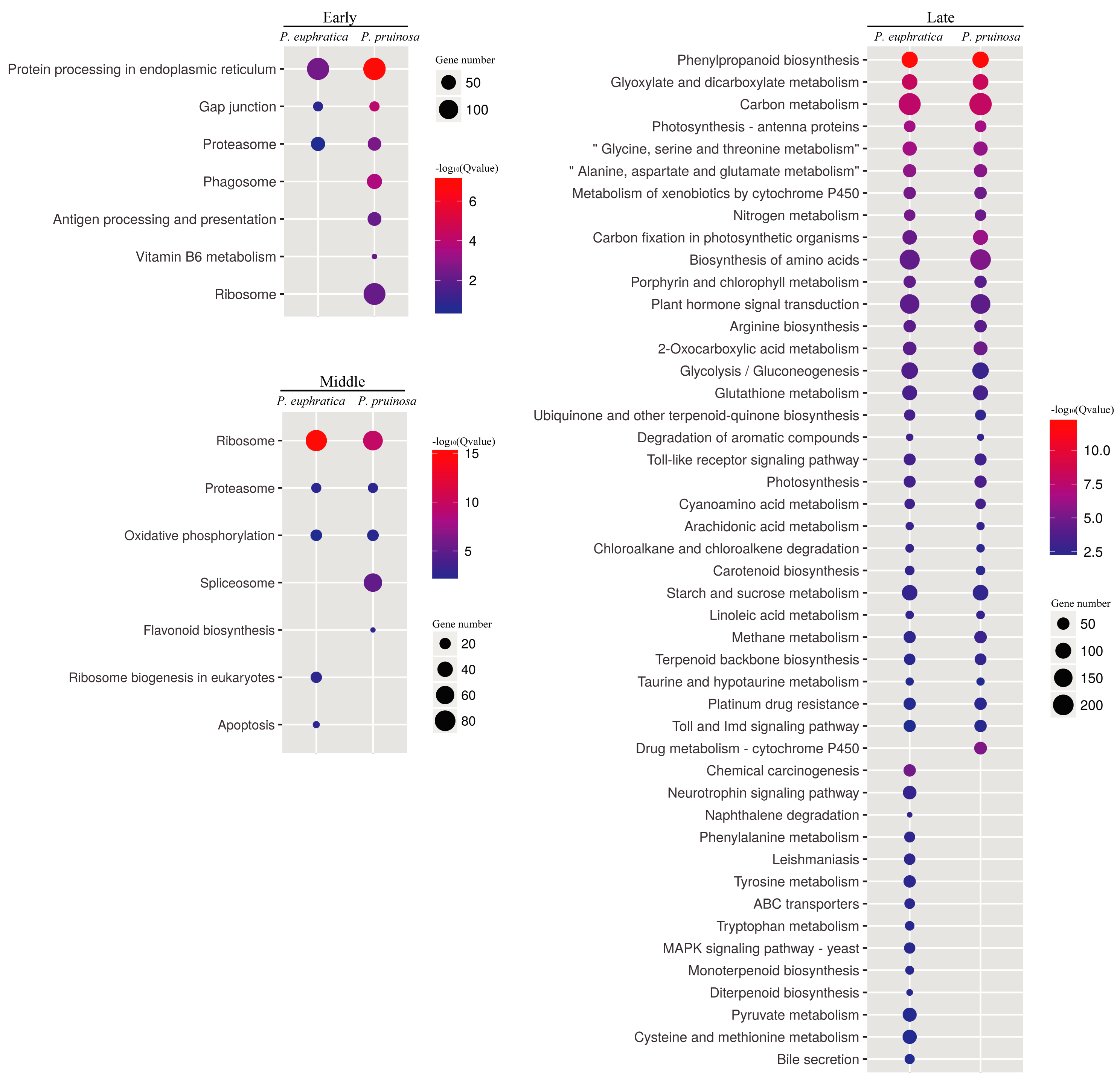

Supplement: FIGURE S6 — KEGG function enrichment of the DEGs for seed germination processes. [file Image_6.tif]

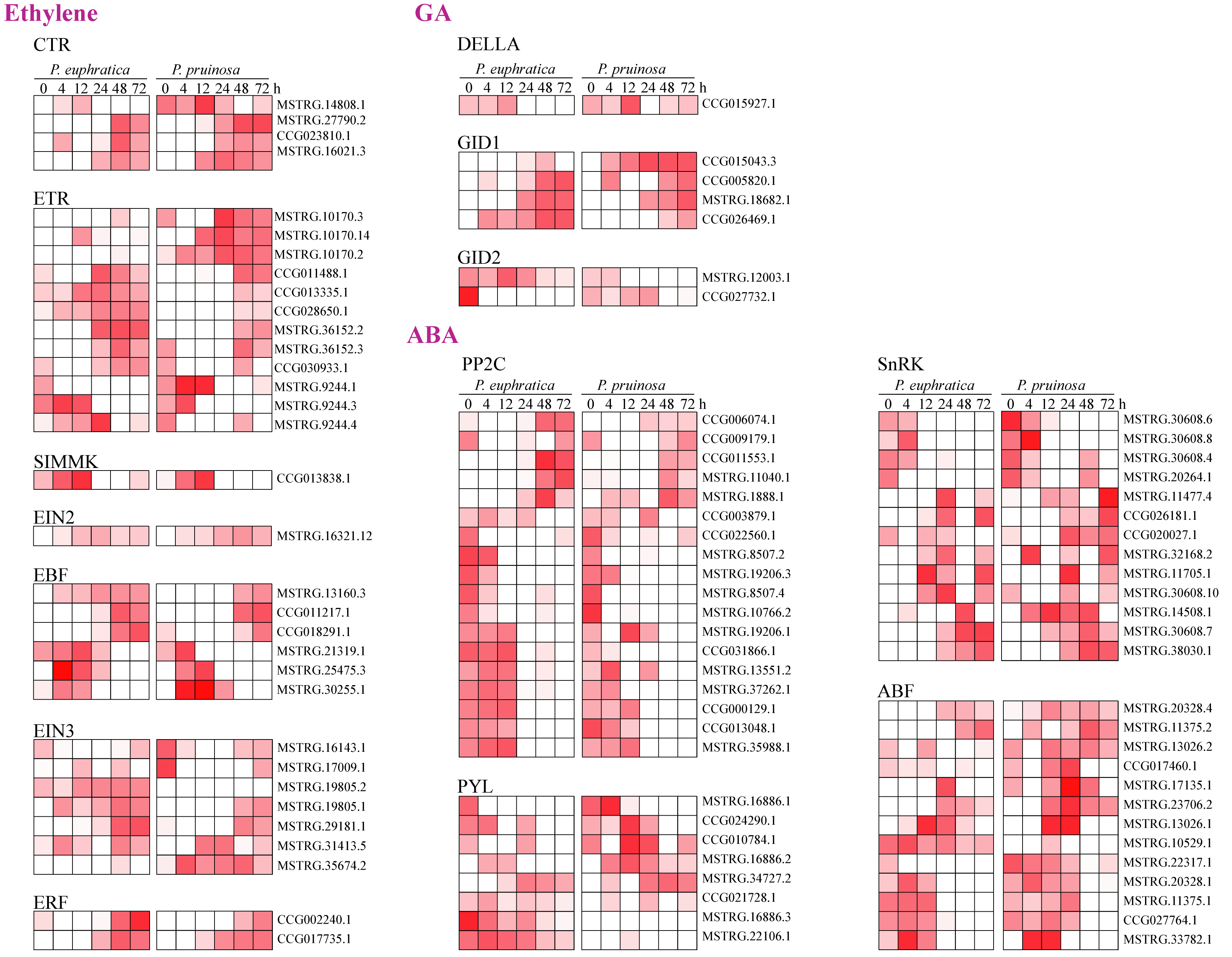

Supplement: FIGURE S7 — The expression pattern of the hormone-related genes in the two poplars. Expression patterns of hormone-related genes. Normalized expression levels of genes related to ethylene, GA and ABA are shown. [file Image_7.tif]

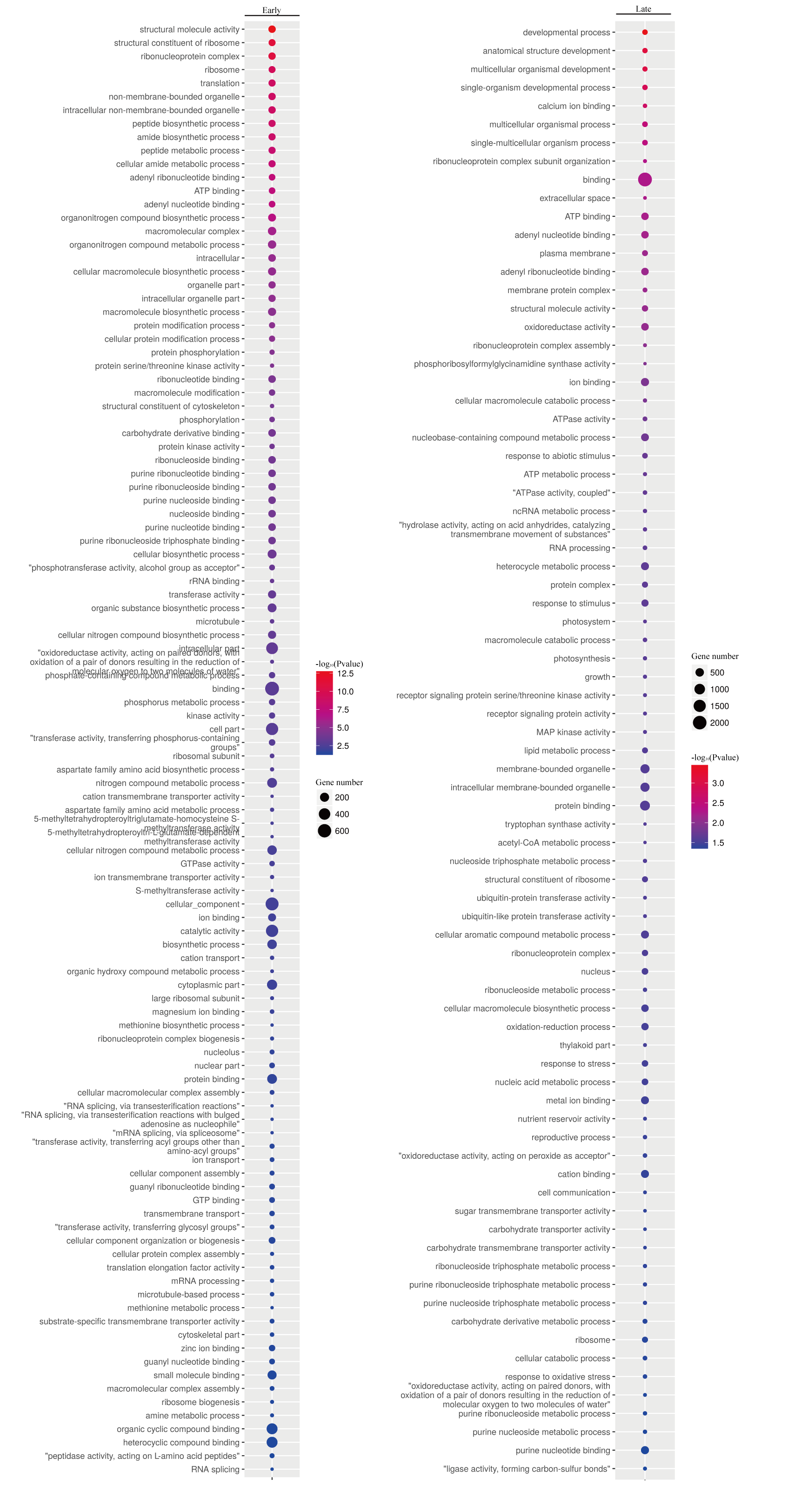

Supplement: FIGURE S8 — GO function enrichment of the DEGs for salt tolerance variety of the two species. [file Image_8.TIF]

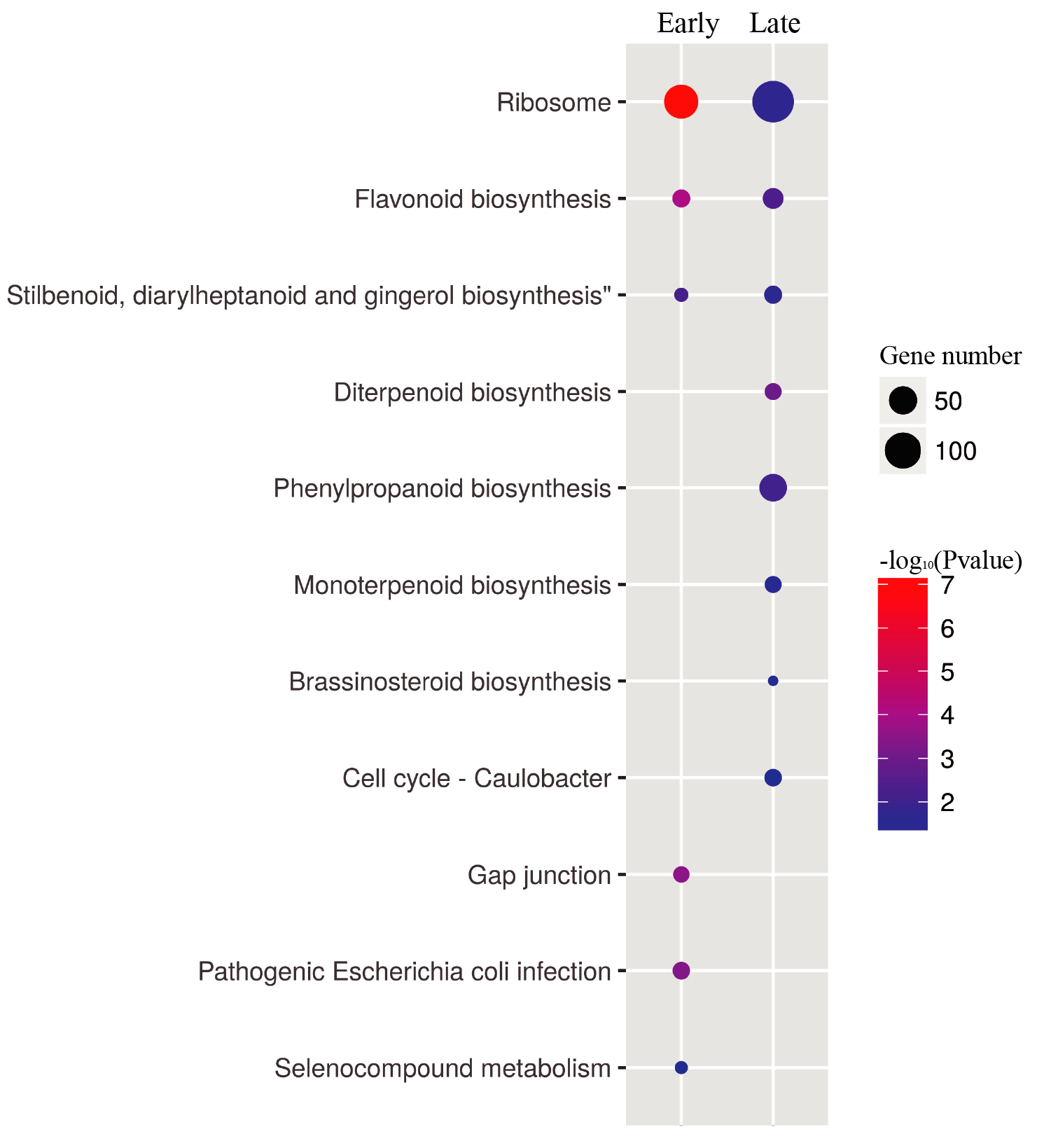

Supplement: FIGURE S9 — KEGG function enrichment of the DEGs for salt tolerance variety of the two species. [file Image_9.TIF]
