# Supplementary material for: Transcriptomic Analysis of Seed Germination Under Salt Stress in Two Desert Sister Species (Populus euphratica and P. pruinosa)
Source: Front Genet. 2019 Mar 25;10:231. doi: 10.3389/fgene.2019.00231 (PMC6442517; doi:10.3389/fgene.2019.00231)
Supplement: TABLE S2 — Summary of the illumine sequencing reads and the matches in the P. euphratica and P. pruinosa. [file Table_2.DOCX]

| Samples | Uniquely aligned reads (%) | Multimapped reads (%) | Overall alignment rate (%) | Samples | Uniquely aligned reads (%) | Multimapped reads (%) | Overall alignment rate (%) |
| --- | --- | --- | --- | --- | --- | --- | --- |
| Peu-0h-1 | 84.39 | 2.77 | 89.92 | Ppr-0h-1 | 77.11 | 6.00 | 85.41 |
| Peu-0h-2 | 82.58 | 3.07 | 88.50 | Ppr-0h-2 | 77.24 | 5.98 | 85.50 |
| Peu-0h-3 | 82.96 | 3.12 | 88.78 | Ppr-0h-3 | 78.56 | 5.91 | 86.65 |
| Peu-4h-1 | 80.71 | 2.99 | 86.63 | Ppr-4h-1 | 71.44 | 5.27 | 79.96 |
| Peu-4h-2 | 78.34 | 3.37 | 85.01 | Ppr-4h-2 | 71.38 | 5.76 | 80.35 |
| Peu-4h-3 | 84.56 | 3.37 | 90.29 | Ppr-4h-3 | 70.97 | 5.65 | 80.04 |
| Peu-12h-1 | 81.93 | 3.07 | 87.80 | Ppr-12h-1 | 63.76 | 5.29 | 73.28 |
| Peu-12h-2 | 76.28 | 3.21 | 82.94 | Ppr-12h-2 | 74.69 | 5.69 | 83.28 |
| Peu-12h-3 | 87.30 | 3.58 | 92.64 | Ppr-12h-3 | 64.19 | 5.43 | 73.81 |
| Peu-24h-1 | 86.17 | 3.56 | 91.43 | Ppr-24h-1 | 66.66 | 4.98 | 76.21 |
| Peu-24h-2 | 81.25 | 2.96 | 87.46 | Ppr-24h-2 | 71.50 | 5.21 | 80.71 |
| Peu-24h-3 | 88.40 | 3.28 | 93.18 | Ppr-24h-3 | 75.42 | 5.23 | 84.11 |
| Peu-48h-1 | 87.56 | 3.81 | 92.61 | Ppr-48h-1 | 78.85 | 6.56 | 87.05 |
| Peu-48h-2 | 85.71 | 4.12 | 91.23 | Ppr-48h-2 | 78.52 | 6.71 | 86.90 |
| Peu-48h-3 | 85.44 | 3.88 | 90.85 | Ppr-48h-3 | 78.35 | 6.12 | 86.32 |
| Peu-72h-1 | 87.62 | 3.83 | 92.74 | Ppr-72h-1 | 73.98 | 6.47 | 83.02 |
| Peu-72h-2 | 85.60 | 4.27 | 91.33 | Ppr-72h-2 | 76.02 | 6.19 | 84.41 |
| Peu-72h-3 | 85.76 | 3.58 | 90.80 | Ppr-72h-3 | 78.44 | 6.36 | 86.54 |

Table S2. Summary of the illumine sequencing reads and the matches in the *P. euphratica* and *P. pruinosa*
