# Supplementary material for: Transcriptomic Analysis of Seed Germination Under Salt Stress in Two Desert Sister Species (Populus euphratica and P. pruinosa)
Source: Front Genet. 2019 Mar 25;10:231. doi: 10.3389/fgene.2019.00231 (PMC6442517; doi:10.3389/fgene.2019.00231)
Supplement: TABLE S4 — Primer used for real-time quantitative PCR in this study. [file Table_4.DOCX]

| Gene ID | Annotation | Forward primers(5'-3') | Reverse primers(5'-3') |
| --- | --- | --- | --- |
| CCG025796.1 | ROT3 | TTCCTTTAGGCACTCTTGGATGGC | TCAACTCGGTTAGAGATTTCGGG |
| MSTRG.25895.3 | DWF4 | GAAACGGTCAGGAGAGACGGAAT | GAAGCACCACGAGCATTGTTAT |
| CCG026469.1 | GID1C | GAACCACGGGCCTCCTTAAC | GTCCAGCCATCATCATACGC |
| CCG010784.1 | PYL7 | GAGCAGGTGTGTTATGAACGGAGAG | CCCATCAGGCACATCTACAACGAAT |
| MSTRG.33263.1 | PAL6 | GTATCTTTGGAAATGGCACGGAG | CTGTCAAGAGCCCAGCAATGTAG |
| Actin | CYC063 | CCACGAACGCAGAGAACTT | CTCCATAGATTGATTCTCCTCCG |

Table S4. Primer used for real-time quantitative PCR in this study
